# Dengue Virus Induces the Expression and Release of Endocan from Endothelial Cells by an NS1–TLR4-Dependent Mechanism

**DOI:** 10.3390/microorganisms9061305

**Published:** 2021-06-15

**Authors:** Carlos Alonso Domínguez-Alemán, Luis Alberto Sánchez-Vargas, Karina Guadalupe Hernández-Flores, Andrea Isabel Torres-Zugaide, Arturo Reyes-Sandoval, Leticia Cedillo-Barrón, Ricardo Remes-Ruiz, Héctor Vivanco-Cid

**Affiliations:** 1 Instituto de Investigaciones Médico-Biológicas, Universidad Veracruzana, Iturbide SN. Centro, Veracruz 91700, Mexico; dguezalonso@gmail.com (C.A.D.-A.); kimicoluis@hotmail.com (L.A.S.-V.); karinhernandez@uv.mx (K.G.H.-F.); 2 Facultad de Medicina, Universidad Veracruzana, Atenas y Managua SN. Nueva Mina. Minatitlán, Veracruz 96760, Mexico; andretorres@uv.mx; 3 Nuffield Department of Medicine, The Jenner Institute, University of Oxford, The Henry Wellcome Building for Molecular Physiology, Roosevelt Drive, Oxford OX3 7DQ, UK; arturo.reyes@ndm.ox.ac.uk; 4 Instituto Politécnico Nacional, IPN. Av. Luis Enrique Erro s/n, Unidad Adolfo López Mateos, Mexico City 07738, Mexico; 5 Centro de Investigación y de Estudios Avanzados IPN, Departamento de Biomedicina Molecular, Avenida Instituto Politécnico 2508, San Pedro Zacatenco, Mexico City 07360, Mexico; lcedillo@cinvestav.mx; 6 Hospital Regional de Alta Especialidad, Servicios de Salud de Veracruz, Av. 20 de Noviembre Centro, Veracruz 91700, Mexico; riruiz@yahoo.com.mx

**Keywords:** dengue, endothelial cells, endocan, vascular dysfunction

## Abstract

A common hallmark of dengue infections is the dysfunction of the vascular endothelium induced by different biological mechanisms. In this paper, we studied the role of recombinant NS1 proteins representing the four dengue serotypes, and their role in promoting the expression and release of endocan, which is a highly specific biomarker of endothelial cell activation. We evaluated mRNA expression and the levels of endocan protein in vitro following the stimulation of HUVEC and HMEC-1 cell lines with recombinant NS1 proteins. NS1 proteins increase endocan mRNA expression 48 h post-activation in both endothelial cell lines. Endocan mRNA expression levels were higher in HUVEC and HMEC-1 cells stimulated with NS1 proteins than in non-stimulated cells (*p* < 0.05). A two-fold to three-fold increase in endocan protein release was observed after the stimulation of HUVECs or HMEC-1 cells with NS1 proteins compared with that in non-stimulated cells (*p* < 0.05). The blockade of Toll-like receptor 4 (TLR-4) signaling on HMEC-1 cells with an antagonistic antibody prevented NS1-dependent endocan production. Dengue-infected patients showed elevated serum endocan levels (≥30 ng/mL) during early dengue infection. High endocan serum levels were associated with laboratory abnormalities, such as lymphopenia and thrombocytopenia, and are associated with the presence of NS1 in the serum.

## 1. Introduction

Dengue infections are a current global public health concern worldwide. Four DENV serotypes (DENV1, DENV2, DENV3, and DENV4) circulate in tropical and subtropical areas of the globe, each of which is capable of causing a wide clinical spectrum of disease, including a mild febrile illness known as non-severe dengue. This illness is divided into two different clinical categories (with and without warning signs) and a third category known as severe dengue infection, which is characterized by plasma leakage and fluid accumulation that leads to shock or respiratory distress, severe bleeding, and severe organ impairment [1,2,3]. During the clinical progression of dengue disease, endothelial cells represent a major target of the host innate immune response to viral infection. The proinflammatory response to this viral infection, also known as a “cytokine storm”, acts on the endothelium and promotes cell activation, which plays a major role in DENV-induced vascular permeability [4,5,6]. Direct interactions between endothelial cells and soluble viral antigens, such as nonstructural protein 1 (NS1), in addition to infection with dengue virus particles have been documented [7,8,9,10,11]. NS1 protein is considered one of the most important virulence factors and its biological role in dengue-induced vascular leakage is now being described and investigated in the scientific literature. The NS1 viral protein is continuously secreted by dengue-infected host cells into the serum in hexamer form [12,13]. The serum levels of NS1 in dengue-infected patients are positively correlated with disease severity [14,15]. Two different mechanisms have been described to explain the biological effects of NS1 on endothelial cells. One indirect mechanism involves the disruption of endothelial junctions after the activation of endothelial cells by proinflammatory cytokines, which are mainly produced by NS1-activated peripheral blood mononuclear cells in a TLR4-dependent mechanism [7]. In the second direct mechanism, NS1 causes vascular leakage by inducing endothelial glycocalyx degradation mediated by heparanase-1 (HPA-1) [16,17]. Despite this new in vitro and in vivo evidence of the role of NS1 as a virulence factor with biological effects on vascular endothelial cells, some questions remain to be answered for this particular host–pathogen interaction.

In this paper, we describe for the first time how the NS1 protein in the dengue virus regulates the expression and production of endocan, which is an endothelial cell-specific molecule that has been used as an endothelial cell activation biomarker. Endocan may function as a systemic predictor of severity and fatality in bacteremia models and, in addition, as a diagnostic and prognostic marker for sepsis and pulmonary and cardiovascular diseases [18,19]. Independent of its use as a clinical biomarker, circulating endocan plays a significant role in immunity, inflammation, and endothelial function. The more relevant biological roles of endocan include binding to the leukocyte integrin LFA-1 (leukocyte functional antigen-1) and blocking its interaction with its endothelial ligand, ICAM-1, thus protecting the endothelium from the migration and proliferation of inflammatory cells [20,21]. The upregulation of endocan expression during some viral infections has been recently described for Epstein–Barr virus infections, cytomegalovirus, hepatitis C virus, and Crimean–Congo hemorrhagic fever (CCHF), which is a tick-borne zoonotic viral disease [22,23,24,25].

Using a combination of in vitro (human endothelial cell lines) and ex vivo (measurement of endocan serum levels in dengue-infected patients) approaches, we report, for the first time, the regulation and production of endocan in response to the NS1 protein, which is one of the major virulence factors of dengue virus.

## 2. Materials and Methods

### 2.1. Cell Cultures

The human umbilical vein cell line EA.hy926 was kindly donated by Dra. Rocío Gómez (Facultad de Farmacia, Universidad Autonóma del Estado de Morelos, Mexico) and propagated (passages 5–8) and maintained at 37 °C in humidified air with 5% CO_2_ in DMEM/F12 medium (Sigma, St. Louis, MO, USA) while supplemented with 1% antibiotics and 10% fetal bovine serum (Biowest, Riverside, MO, USA) [26]. The human dermal microvascular endothelial cell line HMEC-1 was kindly donated by Dr. Leticia Cedillo (Departamento de Biomedicina Molecular del Centro de Investigaciones y Estudios Avanzados, Mexico) and propagated (passages 6–10) and maintained at 37 °C in humidified air with 5% CO_2_ in MCDB 131 medium (Sigma Aldrich, St. Louis, MO, USA) supplemented with 10% SFB, 0.2% epidermal growth factor, and 0.4% hydrocortisone [27].

### 2.2. Recombinant NS1 Proteins from Dengue

The recombinant NS1 proteins from DENV1 (strain Nauru/Western Pacific/1974), DENV2 (strain Thailand/16681/84), DENV3 (strain Sri Lanka D3/H/IMTSSA-SRI/2000/1266), and DENV4 (strain Dominica/814669/1981) used in all experiments were produced by Native Antigen (Oxfordshire, UK) in HEK293 cells and were shown to be >95% pure and oligomeric, as is demonstrated by native PAGE and Western blot analyses [16]. In addition, the NS1 proteins were tested and shown to be free of endotoxin contaminants which is determined using the Endpoint Chromogenic Limulus Amebocyte Lysate (LAL) QCL-1000TM kit (Lonza) (<0.1 EU/mL) and as certified by the manufacturer.

### 2.3. Evaluation of Endocan Expression by qPCR Assay

In total, 1 × 10^5^ cells from the HMEC-1 or EA.hy926 cell line were seeded in 12-well plates and incubated with 1 µg/mL of LPS as a positive control or with 5 µg/mL of NS1 protein derived from different dengue serotypes for 24 h, 48 h, and 72 h. We harvested the supernatant and the endothelial cells were collected in TRIzol (Invitrogen, Carlsbad, CA, USA) and kept at 80 °C until assaying. The mRNA from endothelial cells was isolated using TRIzol reagent according to the manufacturer’s suggested protocol. Complementary DNA was elaborated using the SuperScript VILO cDNA synthesis kit (Invitrogen, Carlsbad, CA, USA). The expression of endocan mRNA was quantified using TaqMan probes and the StepOne PCR system, following the 2^−ΔΔCT^ method using β-actin as a housekeeping gene. The Taqman probes for human endocan (Hs00199831_m1) and β-actin (4326315E) were predesigned by Applied Biosystems. The expression of endocan was normalized to the expression of the reference gene.

### 2.4. Blockage of TLR4 on HMEC Cell Line

In total, 1 × 10^5^ HMEC-1 cells were preincubated with 5 µg/mL of anti-TLR4 monoclonal antibody (Abcam, Cambridge, UK, Cat. ab30667) for 1 h before the addition of LPS or NS1 DENV1 [7] and were then stimulated with LPS (Sigma Aldrich, Saint Louis, MO, USA, Cat. L4391) (1 µg/mL) and NS1 (5 µg/mL) for 24 h, 48 h, and 72 h. Endocan levels were quantified using ELISA as previously described.

### 2.5. Subjects

Dengue patients were recruited in the city of Veracruz, Mexico, according to the clinical and laboratory criteria of the World Health Organization 2009 (WHO 2009) [2]. In total, we tested 45 blood samples from patients with non-severe dengue infection with no warning signs (*n* = 16) (no WS) or with warning signs (*n* = 29) (with WS), such as abdominal pain, persistent vomiting, fluid accumulation, mucosal bleeding, lethargy, liver enlargement, and increasing hematocrit with decreasing platelets. All serum samples were frozen at −80 °C until analysis. The samples were collected from the Hospital Regional de Alta Especialidad de Veracruz during outbreaks that occurred between June 2012 and November 2014. During the study period, dengue virus serotypes 2 and 4 circulated in Veracruz city. A single blood sample was collected from each patient. Based on the time of clinical illness, we classified dengue patients into febrile (1–3 days after disease onset), defervescence (4–7 days after disease onset), and convalescence (samples collected on days 8–10 after disease onset). All the dengue patients showed laboratory evidence of thrombocytopenia (<100,000 counts/mm^3^). Children under 16 years old and those over 60 years old were excluded in order to render the groups comparable by age. Pregnant women were also excluded from our study. In order to rule out the presence of other associated viral or bacterial infections, further serological tests for endemic pathogens such as *Leptospira*, influenza A, hepatitis A, and *Salmonella* spp. were carried out at the time of admission.

Twenty serum samples from healthy controls (HC) were also tested. The HC were derived from the Centro Estatal de la Transfusión Sanguínea of Veracruz in the same geographical region as the dengue patients. The HC had shown no clinical signs or manifestations suggestive of dengue (no febrile symptoms) or other apparent illnesses in the previous 6 months. All HC were negative for dengue in the laboratory tests and tested negative in all infectious disease screening assays performed at the blood bank.

### 2.6. Detection Endocan Production in Supernatant by ELISA

Soluble endocan was measured in the HMEC-1 and EA.hy926 supernatants or in serum samples from patients using a commercial sandwich ELISA kit (Lunginnov, Lille, France) according to the manufacturer’s instructions. Absorbance at 450 nm was determined using an ELISA reader.

### 2.7. Dengue Diagnosis and Laboratory Parameters

In order to confirm the dengue diagnosis, the samples were tested via reverse transcription–polymerase chain reaction (RT-PCR) and using two reference anti-dengue enzyme-linked immunosorbent assays, which are the Dengue IgM (Panbio, Brisbane, Australia) and Dengue IgG (Panbio, Brisbane, Australia). All cases were screened for complete blood counts and liver enzyme levels (glutamic-pyruvic transaminase (GPT) and glutamic oxaloacetic transaminase (GOT)).

### 2.8. Detection of NS1 Antigen in Dengue Patients

The levels of the NS1 antigen were measured in the serum samples with the commercially available diagnostic test SD BIOLINE Dengue Duo (Standard Diagnostic Inc., Gyeonggi-do, Korea) and the results were interpreted in accordance with the manufacturer’s instructions.

### 2.9. Statistical Analysis

The normality of the data distribution was tested using the Kolmogorov–Smirnov test. The data are represented as medians with 25% and 75% interquartile ranges (IQRs) or as means with standard deviations (SDs). The non-parametric Mann–Whitney U test was used to compare two independent groups as appropriate. The Kruskal–Wallis (KW) or analysis of variance (ANOVA) tests were used to compare three or more independent groups where indicated. When the KW or ANOVA tests indicated a statistically significant difference, the Dunn’s or Holm–Sidak multiple comparison tests, respectively, were performed to determine between which groups the differences were sustained. Correlation was evaluated using Spearman’s correlation test. GraphPad Prism (San Diego, CA, USA) version 9 software was used to analyze the data; *p* < 0.05 was considered statistically significant.

## 3. Results

### 3.1. Dengue NS1 Proteins Induce Endocan Expression in Human Endothelial Cells

The NS1 protein has been shown to play a key role in dengue virus pathogenesis by directly triggering endothelial activation and promoting the disruption of endothelial glycocalyx components [16,17,28] as well as by inducing the release of vasoactive cytokines from immune cells [4,5,6,29]. All of these culminated mechanisms have shown a direct correlation with plasma leakage during severe DENV infection [30,31,32,33].

**Figure 1:**
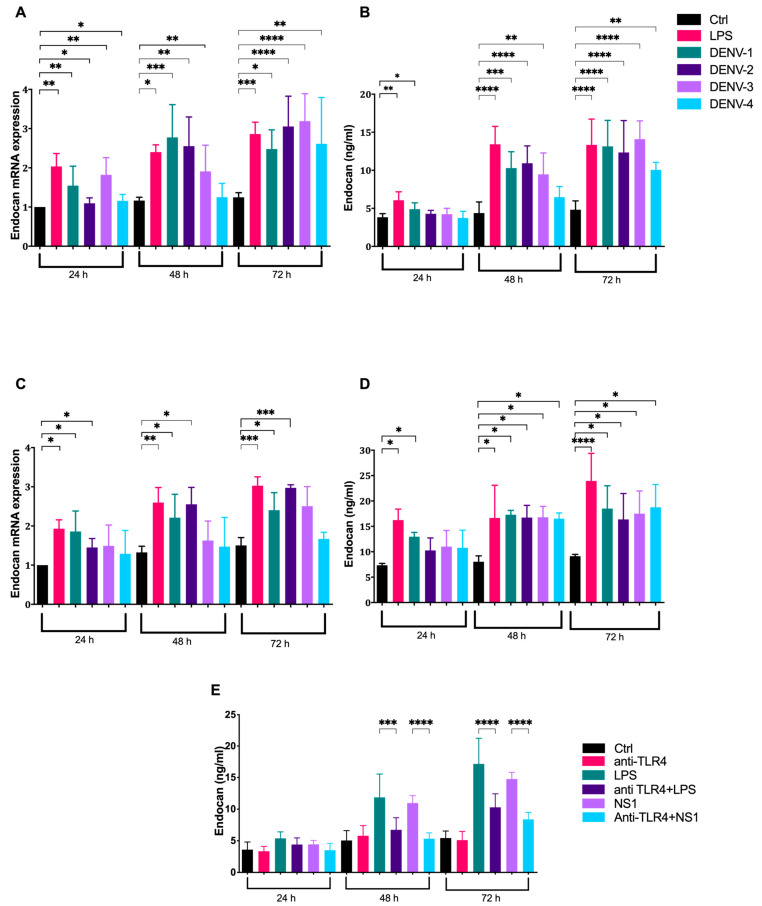
Kinetics of endocan expression and the release induced by NS1 proteins. HMEC (**A**,**B**) and Human umbilical vein cell line, EA.hy926 (HUVEC). (**C**,**D**) Cells were cultured with NS1 derived from the four serotypes (5 μg/mL) for the indicated period and the mRNA expression and serum levels of endocan were then evaluated. HMEC endothelial cells were stimulated with NS1 derived from dengue virus serotype 2 (5 μg/mL), with or without preincubation with the TLR-4 antagonist antibody (5 μg/mL), and the serum endocan levels were then measured (**E**). Experiments were performed in triplicate and repeated three times with similar results. * *p* < 0.05, ** *p* < 0.01, *** *p* < 0.001, **** *p* < 0.0001.

In order to gain further understanding of the role of NS1 in regulating the expression of endocan, qPCR analysis of a key endothelial glycocalyx (EG) gene and the endocan expression in NS1-activated human endothelial cell lines was performed. We measured the endocan mRNA levels in two different endothelial cell lines, HMEC and HUVEC, in the presence of recombinant NS1 proteins from the four serotypes of dengue virus. Endocan mRNA levels in the HMEC cell line showed a marked two-fold to three-fold increase compared to negative controls 48–72 h post-activation with the recombinant NS1 proteins (Figure 1A). In agreement with the above data, the expression of endocan was also increased by two-fold to three-fold in HUVEC cells upon stimulation with NS1 from the Den-1 and Den-2 serotypes after 24–72 h. The mRNA expression showed an increasing trend after the activation of HUVEC cells with NS1 proteins from Den-3 and Den-4 serotypes after 48 h and 72 h, but this was not statistically significant (Figure 1C). In order to investigate whether NS1 can simultaneously induce the production and release of endocan at the protein level in vitro, we measured endocan levels in the supernatants of NS1-stimulated HMEC and HUVEC using a commercial ELISA kit. In the non-stimulated control cells, endocan expression levels were low (Figure 1B,D). After 48 h and 72 h, the expression of endocan in the HMEC cell line stimulated with different NS1 proteins significantly increased relative to that in non-stimulated cells (Figure 1B). Consistent with the above data, the levels of endocan were also significantly increased in the HUVEC cell line after 48 h and 72 h of stimulation with different NS1 proteins (Figure 1D). Taken together, our data demonstrate that endocan expression is induced by NS1 in both human endothelial cell lines in a time-dependent manner.

### 3.2. Dengue NS1 Protein Induces Endocan Expression in Human Endothelial Cells by a Toll-Like Receptor-4-Dependent Mechanism

Previously, it has been shown that endocan expression can be induced by LPS, a very potent bacterial proinflammatory molecule recognized by Toll-like receptor-4 (TLR4) [18,34]. The expression and localization of TLR4 on cell surfaces of primary human endothelial cells or human cells of endothelial origin have already been demonstrated [35,36]. Additionally, NS1 has been shown to induce the production and release of proinflammatory cytokines from peripheral blood mononuclear cells (PBMCs) via the activation of TLR4 [7]. Thus, we examined the potential direct effects of NS1 derived from the dengue virus on the expression of the endocan molecule in human endothelial cell lines through a TLR4-dependent mechanism. We used an antagonist monoclonal anti-TLR4 antibody to block TLR4 activation in vitro, as has previously been described.

HMEC endothelial cells were stimulated with NS1 derived from the dengue virus serotype 2 (5 μg/mL) with or without preincubation with the TLR-4-antagonist antibody. As Figure 1E shows, the concentration of endocan was significantly reduced in the supernatant of cells incubated with the anti-TLR4 antibody and stimulated with NS1, when compared with cells untreated with the anti-TLR4 antibody but stimulated with NS1 (mean endocan concentration in NS1-treated cells: 10.97 ng/mL; endocan concentration in NS1+TLR4mAb: 5.33 ng/mL; *p* < 0.0001). Thus, our results show that the anti-TLR4 antibody efficiently inhibited in vitro endocan expression resulting from the stimulation with NS1 protein.

### 3.3. High Serum Levels of Endocan Are Associated with Clinical Biomarkers of Dengue Infection

**Figure 2:**
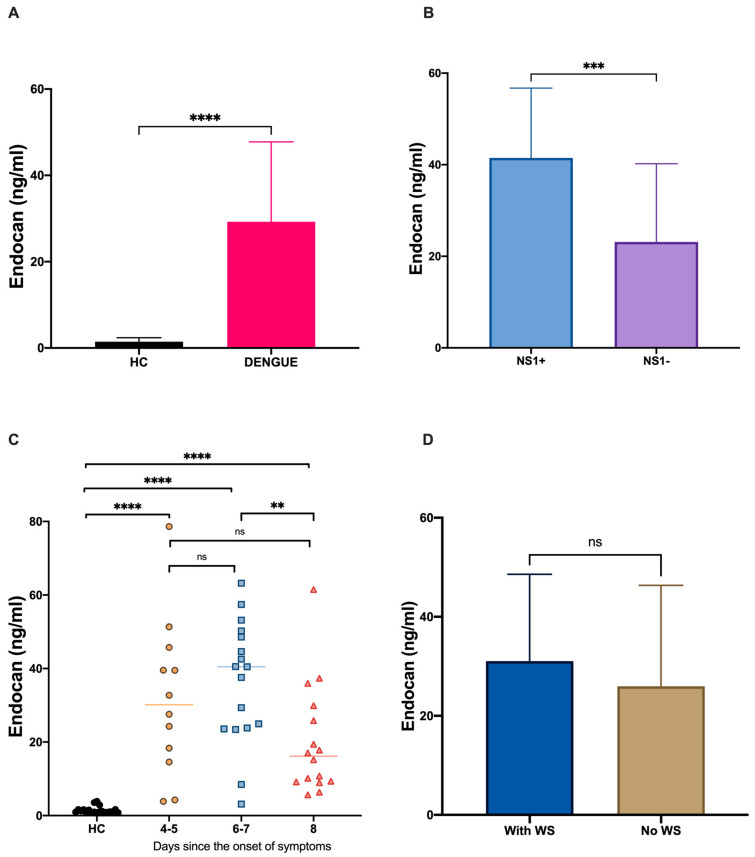
Endocan levels in patients with dengue infection. The serum levels of endocan were evaluated in healthy controls (HC) (*n* = 20) and dengue patients (**A**) (*n* = 45). Dengue patients were classified as positive (*n* = 15) or negative (*n* = 30) for the NS1 antigen (**B**) and were categorized by days after the onset of symptoms (**C**) and as dengue with (With WS) (*n* = 29) or without warning signs (No WS) (*n* = 16) according to the WHO 2009 dengue classification (**D**). The endocan levels were compared in all groups. Data were analyzed using Mann–Whitney’s U test. ** *p* < 0.01, *** *p* < 0.001, **** *p* < 0.0001, ns: not significant.

Given our previous in vitro results, we decided to evaluate the circulating endocan serum levels in dengue-infected patients and their potential correlation with some of the most important biomarkers in the clinical course of dengue infection. A total of 45 dengue-infected patients were recruited; their baseline clinical characteristics and laboratory data are summarized in Table 1. The inclusion criteria were laboratory-confirmed dengue virus (DENV) cases who had been enrolled up to 8 days after the onset of symptoms, which encompasses the acute phase of the disease. Following laboratory evaluations, the medians of the hematological parameters’ values were found to be significantly lower in dengue-infected patients than when compared to healthy controls (Table 1). The patients with non-severe dengue were placed into two different diagnostic groups—dengue without warning signs (D − W) and dengue with warning signs (D + W)—according to the clinical and laboratory criteria of the World Health Organization 2009 [2] (Table 2). The serum levels of endocan in the peripheral blood of DENV-infected patients were measured. The results showed higher levels of endocan here than in healthy controls (Figure 2A). No differences in endocan serum concentration were observed between patients with dengue without warning signs (D − W) and those with dengue with warning signs (D + W). (Figure 2D).

### 3.4. Dengue NS1 Positivity and Endocan Serum Levels

As our in vitro results showed the direct effect of NS1 on endocan expression in human endothelial cells, we decided to analyze and compare endocan serum levels in those who were NS1 antigen-positive against NS1-negative patients. As is shown in Figure 2B, a significant difference in endocan concentration was found between the groups and higher Endocan levels were observed in the NS1 antigen-positive group (NS1+ median 44.61 pg/mL vs. NS1− median 18.88 pg/mL; *p* < 0.0005).

### 3.5. Kinetics of Endocan Serum Levels in the Clinical Course of Dengue Infection

In order to better understand the in vivo regulation and synthesis of endocan over the course of dengue infection, we classified all recruited patients in terms of days since the onset of symptoms (Group 1 = 4–5 days, Group 2 = 6–7 days, and Group 3 = 8 days) and we performed a comparative analysis of blood endocan concentrations between the groups over time. The serum levels of endocan were significantly increased in patients 4–5 days after symptom appearance (*n* = 12, median = 30.14 pg/mL, and SD = 21.28 pg/mL) and the peak corresponding to maximal endocan concentration was found in patients 6–7 days after the onset of symptoms (*n* = 17, median = 40.49 pg/mL, and SD = 16.73 pg/mL). On day 8 after the onset of dengue infection, the endocan concentration in patients was reduced, but the concentration was still higher than the baseline level found in healthy controls (*n* = 16, median = 16.14 pg/mL, and SD = 15.00 pg/mL) (Figure 2C).

### 3.6. Correlation between Endocan Serum Levels and Hematological and Biochemical Laboratory Parameters in Dengue Infection

**Figure 3:**
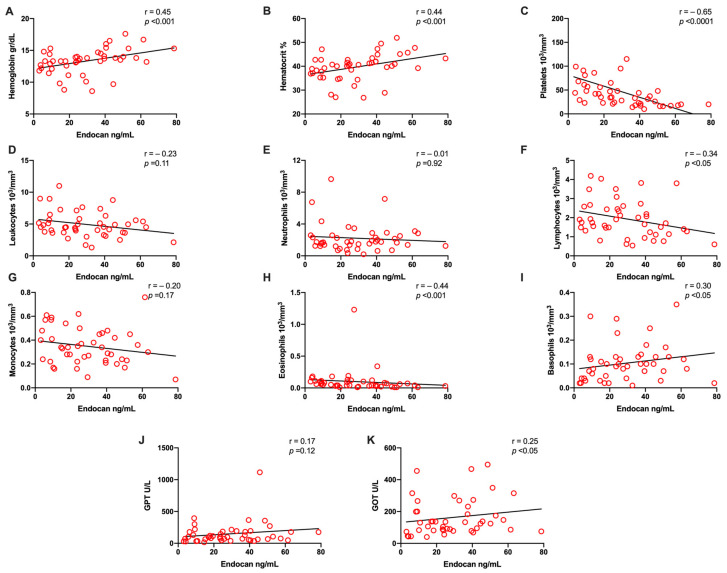
Correlation between Endocan serum levels and hematological and biochemical laboratory parameters in dengue infection. Correlations between endocan serum levels and hemoglobin (**A**), hematocrit (**B**), platelet (**C**), leukocyte (**D**), neutrophil (**E**), lymphocyte (**F**), monocyte (**G**), eosinophil (**H**), basophil (**I**), GPT (**J**), and GOT levels (**K**) were analyzed.

The correlations between serum endocan levels and other laboratory parameters were evaluated. Figure 3 shows that the serum endocan levels were inversely correlated with the lymphocyte, platelet, and eosinophil counts. A positive correlation was observed between serum endocan level and hemoglobin, hematocrit, basophils, lymphocytes, and GOT (Figure 3A,B,F,I,K).

## 4. Discussion

In order to further understand the role of NS1 in the pathogenesis of endothelial cell dysfunction, we performed qPCR analysis of endocan gene expression and immunoassays estimating endocan protein levels in two different NS1-activated endothelial cell lines. Endocan is a soluble dermatan sulfate proteoglycan that is expressed and secreted by the vascular endothelium. We found that the NS1 protein derived from dengue virus upregulates the expression of the endocan gene and promotes endocan protein synthesis in human endothelial cells in a time-dependent manner. In addition, we found that the DENV2 NS1 protein induces endocan expression in the HMEC-1 cell line in a Toll-like receptor-4-dependent mechanism. Previously, it has been shown that TLR4 is expressed on the cell surfaces of HMEC-1 and HUVEC cells [37]. Our results show that anti-TLR4 mAb blocked NS1-induced endocan production. One limitation in our study at this time was the impossibility to determine a direct activation of TLR4 signaling pathways after NS1 stimulation of endothelial cells. Some experiments about this particular issue will be addressed in prospective studies. In our experiments, we used highly purified commercial recombinant NS1 proteins of dengue virus that had been expressed in HEK293 cells, which were certified by the manufacturer as free of endotoxin contaminants, and these NS1 proteins were tested in our laboratory for LPS contamination using the Endpoint Chromogenic Limulus Amebocyte Lysate (LAL) QCL-1000TM kit (Lonza), which confirmed that the samples were endotoxin-free. Our findings reveal that NS1 signaling in HMEC is specifically mediated by TLR4. The latent membrane protein 1 (LMP1) from the Epstein–Barr virus (EBV) has previously been described as an endocan-inducing molecule of viral origin. To the best of our knowledge, this is the second report showing that endocan expression can be regulated by virus-encoded proteins other than LMP1. The upregulation of endocan expression has been recently described in infections with the Epstein–Barr virus, cytomegalovirus, hepatitis C virus, and Crimean–Congo hemorrhagic fever (CCHF), which is a tick-borne zoonotic viral disease [22,23,24,25]. Endocan has also been studied as a novel endothelial cell dysfunction marker in patients with sepsis, severe sepsis, and septic shock [18]. A recent study performed in adults with dengue in Indonesia studied the plasma levels of Endocan and other glycocalyx proteoglycan, such as syndecan [38]. In this study, the authors found that plasma endocan levels were higher during the critical phase in dengue with severe plasma leakage vs. no plasma leakage. However, endocan levels reported in that study are lower than the endocan levels found in our patient cohort. Some possible explanations relevant to these differences could be that infecting dengue serotype, primary or secondary infections, and genetic background and age of studied patients.

Recently, a significant relationship between serum endocan levels with mortality and the need for intensive care in COVID-19 patients was described and it suggested that baseline serum endocan levels may prove useful as prognostic biomarkers in patients hospitalized with COVID-19 [39]. Here, we have shown that the serum levels of endocan increase significantly throughout the clinical course of non-severe dengue infection, with the maximal endocan concentration being reached in patients 6–7 days after the onset of symptoms. Our results suggest that endocan has potential use as a new endothelial activation biomarker in dengue infections. Our study is limited by the fact that we were not able to include severe dengue cases, which is important for establishing whether endocan has any clinically relevant predictive role during the clinical course of severe dengue infection. Interestingly, endocan levels were not significantly elevated in dengue patients with warning signs compared to dengue cases without warning signs, but higher endocan concentrations were found in dengue patients that were positive for the NS1 antigen compared with NS1-negative patients. NS1 circulates during the acute phase of illness in dengue patients and a clinical correlation has been described between the level of secreted NS1, viremia, and disease severity [15]. As NS1 positivity was determined in patients using a qualitative commercial kit at the time of their admission into this study, the circulating NS1 concentration was not included in the analysis of correlation with endocan levels.

On the other hand, correlation analyses between serum endocan levels and hematological parameters were performed in the current study. A negative correlation was observed between the serum endocan level and lymphocyte and platelet counts. By contrast, a positive correlation was observed between the endocan levels and hemoglobin, hematocrit, and GOT parameters. To the best of our knowledge, this is the first study investigating the correlation of endocan levels with those of hematological and biochemical biomarkers traditionally associated with severe dengue infection. Beyond the endocan’s role as a possible clinical biomarker of dengue infection and its biological role in the immunopathogenesis of this viral disease, its further study is very attractive because endocan may take part in systemic inflammation or endothelial cell activation or may have an anti-inflammatory effect by blocking the recruitment, adhesion, and activation of leukocytes, all of which are take on biological roles previously described in the literature.

Taken together, our results give new insight into the interplay between a viral soluble antigen, such as NS1, and the endothelial response during the acute stage of dengue infection.

## Figures and Tables

**Table 1:** Demographic and clinical characteristics of patients and healthy controls.

Characteristics	HC *n* = 20	Dengue without Warning Signs *n* = 16	Dengue with Warning Signs *n* = 29	*p* Value
Male *n* = (%)	9	8 (50)	9 (31.03)	0.997
Day of onset of symptoms, mean (SD)	NA	3.68 (1.74)	5.55 (2.70)	0.01
Age, mean (SD)	32.4 (13.82)	32.5 (15.09)	34.79 (13.98)	0.77
Secondary infection *n* = (%)	NA	16 (100)	29 (100)	1.00
Positive NS1 *n* = (%)	NA	5 (31.25)	10 (34.48)	0.33
Hemoglobin gr/dl, median (SD)	14.3 (0.96)	13.70 (2.07)	13.35 (1.82)	0.09
Hematocrit %, median (SD)	41.85 (2.72)	39.65 (5.41)	40.70 (5.77)	0.06
Platelets 10^3^/mm^3^, median (SD)	289 (58.55)	32.00 (70.79)	35.00 (26.03)	<0.0001
Leukocytes 10^3^/mm^3^, median (SD)	7.55 (1.27)	1.58(0.78)	2.01 (1.05)	<0.0001
Neutrophils 10^3^/mm^3^, median (SD)	4.75 (1.07)	2.03 (2.30)	1.52 (1.38)	<0.0001
Lymphocytes 10^3^/mm^3^, median (SD)	2.30 (0.68)	1.58 (0.78)	2.01 (1.05)	0.006
Monocytes 10^3^/mm^3^, median (SD)	0.50 (0.12)	0.32 (0.14)	0.34 (0.16)	0.002
Eosinophils 10^3^/mm^3^, median (SD)	NA	0.05 (0.05)	0.07 (0.22)	0.46
Basophils 10^3^/mm^3^, median (SD)	NA	0.06 (0.06)	0.10 (0.08)	0.51

**Table 2:** Prevalence of clinical manifestations by study group.

Signs and Symptoms	Dengue without Warning Signs (*n*/%) *n* = 16	Dengue with Warning Signs (*n*/%) *n* = 29	*p*
Sickness	6/37.5	17/58.6	0.2
Headache	11/68.7	17/58.6	0.5
Myalgia	16/100	22/75.8	0.03
Arthralgia	16/100	22/75.8	0.03
Abdominal pain	0/0	27/93.1	<0.0001
Diarrhea	2/12.5	9/31	0.22
Vomiting	2/12.5	11/37.9	0.07
Gingivorrhagia	2/12.5	8/27.5	0.2
Hepatomegaly	0/0	4/13.7	0.1
